# Clinical profile, diagnostic delay, and genetic make-up of cystic fibrosis in Kashmir, India

**DOI:** 10.4103/0970-2113.80318

**Published:** 2011

**Authors:** Tasaduq Ahmad Mir, Mohd Ashraf, Kaiser Ahmed, Javed Chowdhary, B Rehana, Javid Ahmed

**Affiliations:** *Department of Pediatrics, SKIMS Medical College, Srinagar, India*; 1 *Department of Pediatrics, GB Pant Hospital, GMC, Srinagar, India*; 2 *Department of Pediatrics, GMC, Srinagar, Kashmir 190 001, India*

**Keywords:** Asia, cystic fibrosis, diagnostic delay, ethnology, genetic make-up, Kashmir

## Abstract

**Objectives:**

This observational study was done to describe the clinical profile, and delays in diagnosing cystic fibrosis (CF) disease in Kashmir, India.

**Materials and Methods:**

A total of 6758 patients between the ages of 0 and 19 years were registered over a period of 1 year. Out of these, 150 patients suspected of having CF, on clinical grounds, were subjected to pilocarpine iontophoresis, and later on genetic evaluation. Apart from these specific tests, these patients were subjected to laboratory tests like blood counts, blood sugar, KFT, LFT, pancreatic function test, serum electrolytes, and chloride, urine, throat swab, blood culture, ABG analysis, chest and paranasal X-rays. In addition, sonographic evaluation of abdominal organs was carried out to know the status of internal organs. A polymerase chain reaction (PCR)-based test was used for the identification of CF mutation.

**Results:**

CF was diagnosed in three (0.8%) patients. Median age of presentation of CF was 78 months. Family history suggestive of CF was present in one (33.3%) and consanguinity in three (100%) patients. Common clinical manifestations at the time of presentation included recurrent pneumonia in three (100%), failure to thrive in three (100%), recurrent diarrhea in one (33.3%) patients. General physical examination showed pallor in three (100%), malnutrition in three (100%), and clubbing in two (66.7%) patients. Examination of respiratory tract revealed hyperinflation in two (66.7%), rhinitis in two (66.7%), and creptations in two (66.7%) patients. Sonography of abdominal organs revealed pancreatic cysts in one (33.3%), hyperechoeic and increased liver span in two (66.7%), and small gallbladder in one (33.3%). *Staphylococcus aureus* was cultured from sputum in one (33.3%), pseudomonas aeruginosa in one (33.3%) patients. Delta F508 mutation was present in one (33.3%) patient.

**Conclusion:**

CF may be more common in Kashmir and other parts of Asia, than indicated by our study; diagnosis is often considerably delayed when the disease is identified solely on clinical grounds. It would be advisable to raise the index of suspicion about CF.

## INTRODUCTION

Cystic fibrosis (CF) is a complex recessive disorder caused by mutation in cystic fibrosis transmembrane conductance regulator (CFTR) gene resulting in defective epithelial transport of chloride through CFTR channel.[1] Earlier it was thought that cystic fibrosis is extremely rare in India, but the published reports suggest that cystic fibrosis is probably far more common in people of India/Indian origin than was previously thought but is under diagnosed or missed in majority of cases.[2] Cystic fibrosis is the most common genetic life-shortening disorder in the white population, affecting approximately 1:3000 live births.[3] In Asian Americans, prevalence is 1 case per 31,000 population, while in Asia it is 1 case per 90,000.[4]

The CFTR gene is found at the q31.2 locus chromosome 7. CTFR gene is 2,30,000 base pairs long, and creates a protein that is 1480 amino acids long. The most common mutation, ∆ F508 is a deletion (∆) of three nucleotides that results in a loss of the amino acid phenylalanine (F) at the 508^th^ (508) position on the protein. This mutation accounts for two-thirds of CF cases worldwide and 90% of cases in the United States; however, there are over 1400 other mutations that can produce CF.[5]

The objective of our study was to characterize the clinical presentation in terms of the symptoms, age at diagnosis and morbidity, with the goal to raise the pediatricians’ awareness about CF disease in Kashmir.

## MATERIALS AND METHODS

The present study was conducted over a period of one year from September 2006 to August 2007 in the Department of Pediatrics, G. B. Pant Hospital which is an associated hospital of Government Medical College, Srinagar and a tertiary centre. Patients (0–19 years) having the respiratory and/or gastrointestinal symptoms and features of failure to thrive (FTT) were registered, regardless of their sex, socioeconomic status and demographic profile, excluding foreigners (non-Indians). Out of 6758 patients, 150 suspected cases, after a detailed history and through clinical examination were subjected to sweat chloride test by indigenously developed method for sweat collection and chloride estimation,[6] besides the supportive laboratory tests like blood counts, blood sugar, KFT, LFT, pancreatic function test, *S. electrolytes*, and chloride, urine, throat swab and blood culture, ABG analysis, chest and paranasal X-rays. In addition sonographic evaluation of abdominal organs was carried out to know the status of internal organs. The clinical features kept as criteria for including patients in our study to undergo a sweat chloride test were; muconium ileus, recurrent respiratory tract infections, steatorrhea, malabsorption, rectal prolapse, biliary cirrhosis, portal hypertension, bleeding varices, hypoproteinemia, anasarca, deficiency of vitamin A, D, E, and K, failure to thrive, family history of cystic fibrosis, nasal polyps, and salt craving. The patients with clinical features suggestive of asthma, tuberculosis, lactose intolerance, and congenital heart diseases were excluded from the study. Sweat chloride test was performed in accordance with the indigenously developed method.[6]

Sweat chloride value of >60mEq/l was considered to be abnormal and it was repeated after a weak. Sweat chloride of >60mEq/l in a child on two occasions with clinical features of cystic fibrosis was diagnosed as a case of cystic fibrosis. Normal value being <40 mEq/l. Patients with values between 40 and 60mEq/l (indeterminate) were subjected to repeat sweat chloride test. Positive cases were subjected to chromosomal mutation analysis at All India Institute of Medical Science (AIIMS) New Delhi.

## RESULTS

As depicted in Table 1, three patients in our study were confirmed of having CF disease, measuring an incidence of 1:2,253 (0.04%). Among the patients subjected to pilocarpine iontophoresis (150), males were 82 (54.7%) and females were 68 (45.34%). Majority of the patients in our study had respiratory system involvement and among whom most had recurrent bronchopneumonia (36.7%), recurrent pneumonia (18.7%), recurrent wheeze (29.33%), hemoptysis (0.7%). GIT system involvement was present in 14.00%, failure to thrive in 14.7%, rectal prolapse 1.33%, and salt craving 0.7%. History of consanguinity was present in 59 (39.34%) children. Median age of presentation was 78 months.

**Table 1:** Clinical, laboratory and sonological profile of diagnosed cases

Parameter	%age/ mean	Patient I	Patient II	Patient III
Initial sweat chloride level	80.3	71	81.6	88.3
Repeat sweat chloride level	114.3	112.0	114.7	116.3
Genetics for ∆F508	33.3	N	N	∆F508/∆F508
Sex (M:F)	1: 2	M	F	F
Age (months) at presentation	78	60	156	18
Recurrent chest infection	100	P	P	P
Failure to thrive	100	P	P	P
Recurrent diarrheas	33.3	A	P	A
Family h/o sib death	100	P	P	P
CF in sib/s	33.3	A	P	P
H/o consanguinity	100	P	P	P
Pallor	100	P	P	P
Malnutrition	100	P	P	P
Clubbing	66.7	P	P	A
B/L infiltrations on X-ray	66.7	A	P	P
Mid-zone consolidation	33.3	A	A	P
P/o crepitations and wheez	66.7	A	P	P
*S. aureus* on sputum culture	33.3	P	A	A
*P. aeuroginosa* on sputum culture	33.3	A	P	A
Hyperechoic liver and pancreas on USG abdomen	66.7	P	P	A
Small gall bladder on USG	33.3	P	A	A
Mortality	33.3	P	A	A

P: Present, A: Absent, M: Male, F: Female, N: Not matched with existing mutation types.

Maximum amount of sweat that was collected in our study was 444 mg. Four patients had sweat chloride between 40 and 60 mEq/l and on repeat test, they had sweat chloride of <40mEq/l. Three patients had sweat chloride > 60mEq/l on repeat test. One patient was positive for ∆F508 mutation.

## DISCUSSION

Diagnosis of cystic fibrosis in our patients was based on demonstration of elevated sweat chloride on two occasions in all the three cases in the presence of suggestive clinical features, which is in accordance with the International consensus statements for making a diagnosis of cystic fibrosis.[7] Although high chloride values in sweat may be seen in many conditions like malnutrition, untreated hypothyroidism, Addison’s disease, anorexia nervosa, ectodermal dysplasia, atopic dermatitis, fucosidosis, glycogen storage disorder type I, pseudohypoaldosteronism, but all the three patients were diagnosed on the basis of twice elevated sweat chloride, with typical clinical features of cystic fibrosis; hence, chances of false positive were very remote. Consanguinity was present in all the three (100%) patients in our study, contradicting the 19% reported by Kabra *et al*.[8] the reason being that consanguinity is highly prevalent in Kashmir.

In our study, examination of chest revealed hyperinflation in two patients (66.7%), wheezing in two patients (66.7%), and crepitations in two patients (66.7%) as compared to study by Kabra,[8] which showed hyperinflation in 83%, wheezing in 83%, crepitations in 92% patients, and bronchial breathing in 17% of patients. Clubbing was present in two (66.7%) patients as compared to Kabra’s study which indicated clubbing 75% patients.[8]

The median age of presentation of CF screened patients was 108 months among whom, three patients had CF disease, where the diagnosis was delayed by 13 ± 3 months. The median age of diagnosis was 78 months in our diagnosed patients. This is higher than average age of presentation than studies done at New Delhi[8] and Chandigarh,[9] where ages of presentation at the time of diagnosis were 54 and 57 months, respectively. This reflects a low index of suspicion for CF in Kashmir leading to delay in diagnosing the disease and raising the morbidity and mortality. In view of morbidity documented in our patients, it can be supposed that many patients might get unnoticed unto their deaths, due to lack of awareness among the treating physicians regarding the prevalence of CF in the state. The clinical condition of two of our patients at the time of diagnosis was sufficiently severe to be life threatening, and one of them later succumbed. It seems reasonable to suspect that many patients might have died without reaching to a proper diagnosis and treatment. This is supported by a study by Doull *et al*. who reported four early deaths among 59 unscreened patients with CF, and no deaths among the 74 screened patients.[10]

Malnutrition was present in all the three (100%) patients, among whom one was severely malnourished and the other two were having mild to moderate malnutrition which can be explained by delay in diagnosing CF and specific intervention. This is contrary to the study done in India[11] but is consistent with the western studies,[12] henceforth needing a stable quantitative and qualitative changes in dietary intake.

In our study, one of the patients was already colonized with pseudomonas aeruginosa (33.3%) and the other one (33.3%) was colonized with *Staphylococcus aureus*, none among them grew *Hemophilus influenzae* and *Klebsella* spp, which was contradictory to the study done by Saiman[13] at the time of diagnosis who reported *Klebsiella* spp and *H. influenza* in good number of patients. Although the incidence of inguinal hernia, hydrocele, and undescended testes is higher than expected in CF patients, was not present in our only male (33.3%) CF patient. In addition, one CF female child who was 13 years had no evidence of secondary sexual characters which was consistent with the studies done in India and the world over.

None of our patients had osteoporosis or diabetes contradicting the studies done in the rest of the world[14,15] which can be explained on the basis of less number of patients and lower age of presentation.

Ultrasonographic findings of our patients showed hyperechogenicity of liver and pancreas in two (66.7%), pancreatic cysts, and small gall bladder in one (33.3%) each, which is lower percentage as reported by Haber,[16] and could be due to early stage of the disease and less number of patients in our study.

Among the three patients diagnosed as CF, only one (33.3%) showed the ∆F508 (homozygous) that is much lower than the 70% reported in Caucasian population.[17] One of our patients who had right lung collapse died during the postoperative period giving a mortality rate of 33.3% but was negative for ∆F508 mutation that goes against the study done by McCormick *et al*., who reported worse prognosis among homogenous ∆F508 mutation of Asian origin.[18] Mutation analysis of our study is showing a 33.3% of ∆F508 (homozygous) mutation well comparable to the other Indian studies.[19] A larger study thus needs to be carried out to identify a panel of common mutations among the Indian CF patients to allow a uniform molecular diagnosis in all clinically suspected cases.

### Limitations

Our study is a regional study and only diseased cases were registered.

All those cases who had mild form of disease and those who died, either before reaching the hospital or because of false diagnosis, did not get registered might project falsely low incidence of our study, apart from our study included only pediatric population (<19 years).
